# Fe effect on the optical properties of TiO_2_:Fe_2_O_3_ nanostructured composites supported on SiO_2_ microsphere assemblies

**DOI:** 10.1186/1556-276X-9-499

**Published:** 2014-09-15

**Authors:** Jesús I Peña-Flores, Abraham F Palomec-Garfias, César Márquez-Beltrán, Enrique Sánchez-Mora, Estela Gómez-Barojas, Felipe Pérez-Rodríguez

**Affiliations:** 1 Instituto de Física, Benemérita Universidad Autónoma de Puebla, Apdo. Post. J-48, Puebla Pue. 72570, México; 2 CIDS-IC, Benemérita Universidad Autónoma de Puebla, Apdo. Post. 196, Puebla Pue. 72000, México

**Keywords:** TiO_2_:Fe_2_O_3_ films, Nanostructured composites, SiO_2_ microsphere assemblies, Sol-gel, Dip coating

## Abstract

The effect of Fe ion concentration on the morphological, structural, and optical properties of TiO_2_ films supported on silica (SiO_2_) opals has been studied. TiO_2_:Fe_2_O_3_ films were prepared by the sol-gel method in combination with a vertical dip coating procedure; precursor solutions of Ti and Fe were deposited on a monolayer of SiO_2_ opals previously deposited on a glass substrate by the same procedure. After the dip coating process has been carried out, the samples were thermally treated to obtain the TiO_2_:Fe_2_O_3_/SiO_2_ composites at the Fe ion concentrations of 1, 3, and 5 wt%. Scanning electron microscopy (SEM) micrographs show the formation of colloidal silica microspheres of about 50 nm diameter autoensembled in a hexagonal close-packed fashion. Although the X-ray diffractograms show no significant effect of Fe ion concentration on the crystal structure of TiO_2_, the μ-Raman and reflectance spectra do show that the intensity of a phonon vibration mode and the energy bandgap of TiO_2_ decrease as the Fe^+3^ ion concentration increases.

## Background

SiO_2_ microsphere assemblies coated with metal oxide films have been considered as promising candidate materials for applications in photocatalysis due to their large surface area, excellent accessibility to the inner surface, and suitable morphology in comparison with powder materials. For example, mesoporous TiO_2_ films perform quite well in the decomposing of pollutants and in the generation of hydrogen by water splitting [1]. Also, the TiO_2_ is the most promising catalyst due to its high efficiency, stability, and low cost. It has been used widely in the photocatalytic degradation of phenol. However, one disadvantage of TiO_2_ for industrial applications is the need of filtration after the photodegradation process. Recently, the research has been focused on modifying the surface or the bulk of a semiconductor catalyst by adding transition metal impurities, which give rise to the mixed oxide semiconductor formation [2].

However, few research studies have been concerned to surface wetting application. One interest point in our current research is to observe the effect of light adsorption on surface materials with respect to their wetting properties particularly of the Fe_2_O_3_:TiO_2_/SiO_2_ composites; this topic has attracted significant scientific attention particularly for biological systems. A method for the fabrication of thin films using sol-gel is the dip coating procedure, and it is an appropriate technique to obtain TiO_2_[3], Fe_2_O_3_:TiO_2_[4], and TiO_2_/SiO_2_[5], among others. This method has some advantages, for example, low investment cost for production facilities, large variety of coating materials, and high uniformity on large coating areas. Furthermore, an additional advantage of this technique is the fact that both sides of the substrate can be coated simultaneously. The versatility of this technique allows us to control the vertical dipping velocity, which is very important in the wetness of the forming layer and in the gelation of the layer by the solvent evaporation in order to get uniformity in the film thickness. The *α*-Fe_2_O_3_ has the important feature of absorbing a large part of the visible solar light due to its energy bandgap of 2.1 eV [6]. Its chemical stability, no toxicity, abundance in nature, and low cost-effective features make it a good material candidate for applications in many fields.

The goal of this work is to synthesize a set of TiO_2_, Fe_2_O_3_, and TiO_2_-Fe_2_O_3_ thin films deposited on SiO_2_ microsphere assemblies by the sol-gel method in combination with a controlled vertical dip coating procedure. Mainly, we have analyzed the effect of Fe ion concentration on the structural and optical properties of these composites.

## Methods

Tetraethylorthosilicate (TEOS, 99%), ammonium hydroxide (28%), and titanium(IV) buthoxide (97%) were purchased from Sigma-Aldrich (St. Louis, MO, USA); ethyl alcohol (99.5%), iron nitrate nonahydrate (99.8%), hydrochloric acid (38%), and monoethanolamine (99.4%) were purchased from J.T.Baker (Center Valley, PA, USA); 2-methoxyethanol was supplied by Fluka (St. Louis, MO, USA). All chemicals were used without additional purification. In all experiments, we have used ultrapure water (Easy Pure II System, Thermo Fisher Scientific, Waltham, MA, USA).

The synthesis of SiO_2_ microparticles was carried out by the Stöber method. Following this method, it is possible to control the diameter of the spheres from the TEOS concentration. First, we made a solution by mixing ammonium hydroxide, ethanol, and water with volumes of 20, 38.4, and 41.6 ml, respectively. Then, we made a second solution with 6.6 ml of TEOS and 93.4 ml of ethanol, and later on, we mixed both solutions and stirred it for 1 h at room temperature. The TiO_2_ precursor solution was made by mixing and stirring the following chemical compounds for 2 h: 19.2 ml of ethanol, 3.8 ml of hydrochloric acid, 7.7 ml of water, and 19.2 ml of titanium(IV) buthoxide at room temperature. The Fe_2_O_3_ precursor solution was obtained as follows: 15.15 g of iron nitrate was dissolved in 60 ml of 2-methoxyethanol and 6.8 ml of monoethanolamine, and this solution was stirred for 2 h at room temperature. Finally, to have solutions to the 1, 3, and 5 wt% of Fe^3+^ with respect to the TiO_2_ precursor solution, 0.0331, 0.0995, and 0.1658 g of iron nitrate, respectively, were required.

Once the SiO_2_ spheres have been obtained, a solution with 20 wt% of SiO_2_ microspheres was prepared and impregnated on a glass substrate by the dip coating procedure at 3 mm/min rate. The glass substrate with the SiO_2_ spheres was immersed into the corresponding TiO_2_ precursor solution using the same vertical dip coating procedure at 1.5 mm/min rate. All immersion procedures were done at room temperature and normal pressure. Then, the sample containing the TiO_2_ coating was annealed at 500°C with air flux 1 ml/s for 6 h.

The Fe_2_O_3_ coating of the SiO_2_ opals was obtained by following the same dip coating procedure with the Fe precursor solution under the same conditions. Finally, the TiO_2_:Fe_2_O_3_ (1, 3, 5 wt%) films supported on the SiO_2_ opals were obtained by adding the appropriate amounts of ferric nitrate nonahydrate into the TiO_2_ precursor solution, and the same dipping procedure was carried out.

The samples were characterized by the technique: X-ray diffraction (XRD; D8 Discover CuK_α_ (*λ* = 1.5406 Å), Bruker AXS, Inc., Madison, WI, USA), μ-Raman spectroscopy (RS; Horiba Jobin Yvon Lab Ram HR, HORIBA, Ltd., Kyoto, Japan), and diffuse reflectance spectroscopy (Varian Cary 100, Agilent Technologies, Inc., Santa Clara, CA, USA). The surface morphology of the samples was studied with a scanning electron microscope (JEOL JSM-6610LV, JEOL Ltd., Akishima-shi, Japan).

## Results and discussion

**Figure 1:**
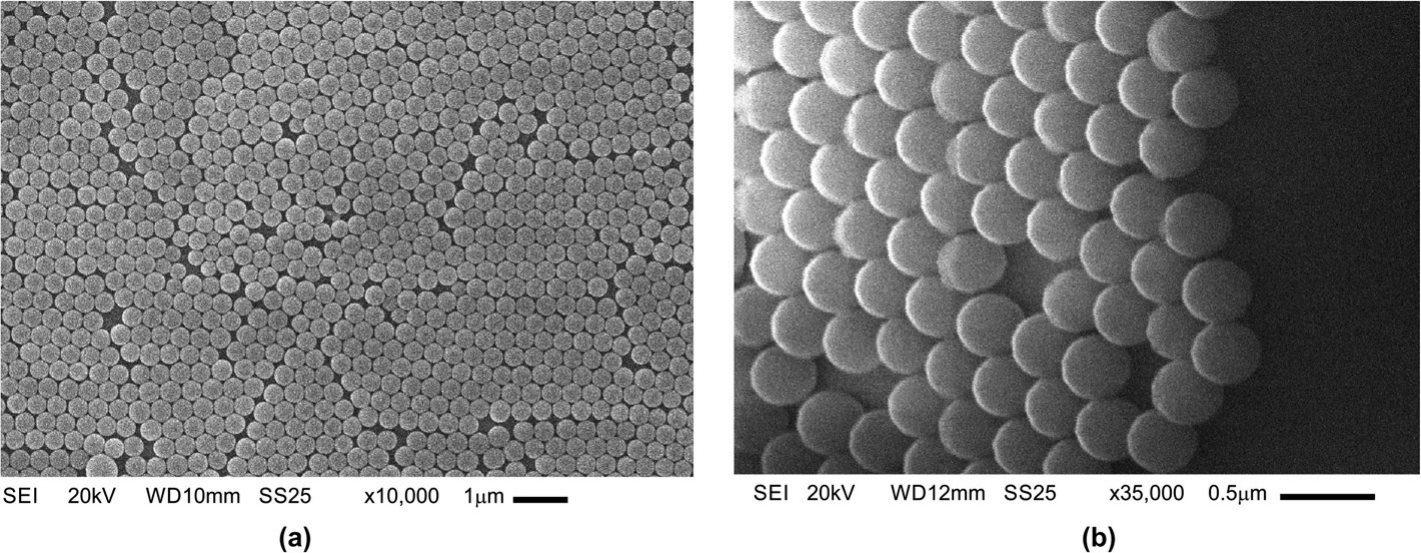
**SiO**_**2 **_**microspheres of 365 nm average size deposited on glass substrate. (a)** SEM micrograph at ×10,000 and **(b)** lateral view of the SiO_2_ spheres at ×35,000 showing the formation of a single layer.

Figure 1a shows a scanning electron microscopy (SEM) micrograph at ×10,000 of the surface morphology of the SiO_2_ microspheres deposited on a glass substrate. It is seen in this figure that in some regions, the SiO_2_ microspheres form a close-packed arrangement, and in others, some fissures are present.

Figure 1b shows a lateral view at ×35,000 of the SiO_2_ microparticles. It is seen that the SiO_2_ spheres are smooth surfaces and their average size is about 365 nm. It is seen also that the SiO_2_ sphere assembly was obtained in a single-layer form; each sphere is surrounded by six nearest spheres in such a way that this arrangement is the most dense of identical circles packed in a plane. The formation of this assembly is mainly due to interparticle repulsion and particle-water interaction. Along the surface, there are fissures suggesting that the packing is not completely homogeneous; this ruptures on the surface are probably produced by the deposition silica method, that is, the glass substrate is immersed into an aqueous solution where the silica microparticles are dispersed and thus the hexagonal closed packed is not very well controlled.

Guo et al. [7] described a controllable method to fabricate hexagonal close-packed Langmuir-Blodgett silica particulate monolayers modifying the silica surface by using a binary surfactant and solvent systems, reducing in this way fissures along the surface layer. The difficulty of this method to be applied in our work is that the TiO_2_ adsorption on the silica-modified surface is not possible due to the hydrophilic character of TiO_2_ and the hydrophobic character of the silica.

**Figure 2:**
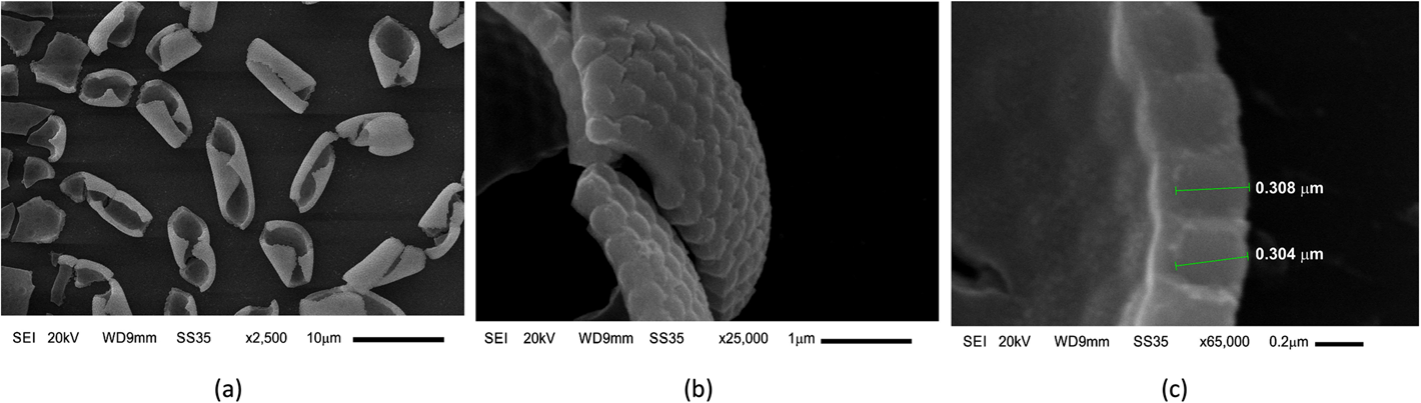
**A SiO**_**2 **_**microsphere layer coated with TiO**_**2**_**:Fe**_**2**_**O**_**3**_**. (a)** Formation of ‘micro-shavings’ (×2500) dispersed on the glass substrate, **(b)** lateral view at ×25,000 of the SiO_2_ spheres on glass substrate, and **(c)** lateral view at ×65,000 of the SiO_2_ spheres on glass substrate.

**Figure 3:**
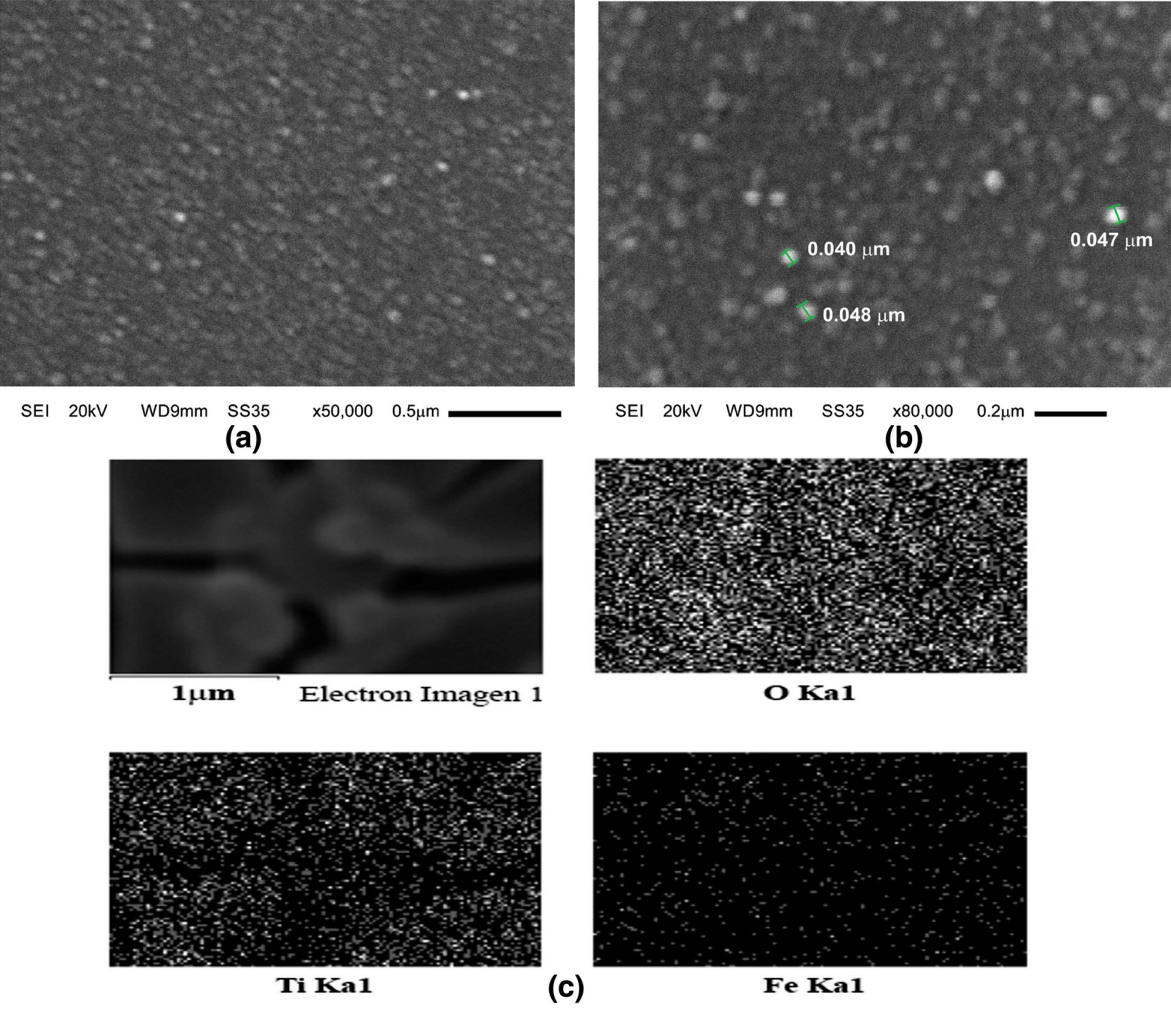
**SEM images of the surface morphology of the TiO**_**2**_**:Fe**_**2**_**O**_**3 **_**composite supported on a SiO**_**2 **_**microsphere layer.** SEM images at **(a)** ×50,000, **(b)** ×80,000, and **(c)** EDS mapping distribution of elements of the TiO_2_:Fe_2_O_3_/SiO_2_ composite: The upper left image corresponds to the mapping region; the upper right corresponds to the oxygen element; the lower left corresponds to the titanium element and the lower right image corresponds to the iron element.

Figure 2 shows SEM images of the SiO_2_ microspheres coated with TiO_2_:Fe_2_O_3_. In Figure 2a at ×2,500, it is observed the presence of ‘micro-shavings’ dispersed on the glass substrate. This is due to the incorporation of TiO_2_:Fe_2_O_3_ coating, which destabilizes the SiO_2_ close-packed arrangement as shown in Figure 1. In Figure 2b, a lateral view at ×25,000 shows with more detail the curving of the SiO_2_ single layer. In Figure 2c, a micrograph at ×65,000 is shown. The SiO_2_ microspheres are peeled off; thus, the image shows an inverse opal like showing that the TiO_2_:Fe_2_O_3_ composite has covered completely the SiO_2_ surface and the interstitial spaces among spheres as well. Furthermore, Figure 3a,b at 50,000 and 80,000 magnifications, respectively, shows a granular surface morphology of the TiO_2_:Fe_2_O_3_ composite coating, with grain diameter of about 50 nm. In Figure 3c, the mapping distribution of elements of the composite shows the presence of oxygen, titanium, and iron elements. The standardless EDS quantification is 56.78 wt% oxygen, 38.27 wt% silicon, 4.74 wt% titanium, and 0.20 wt% iron.

**Figure 4:**
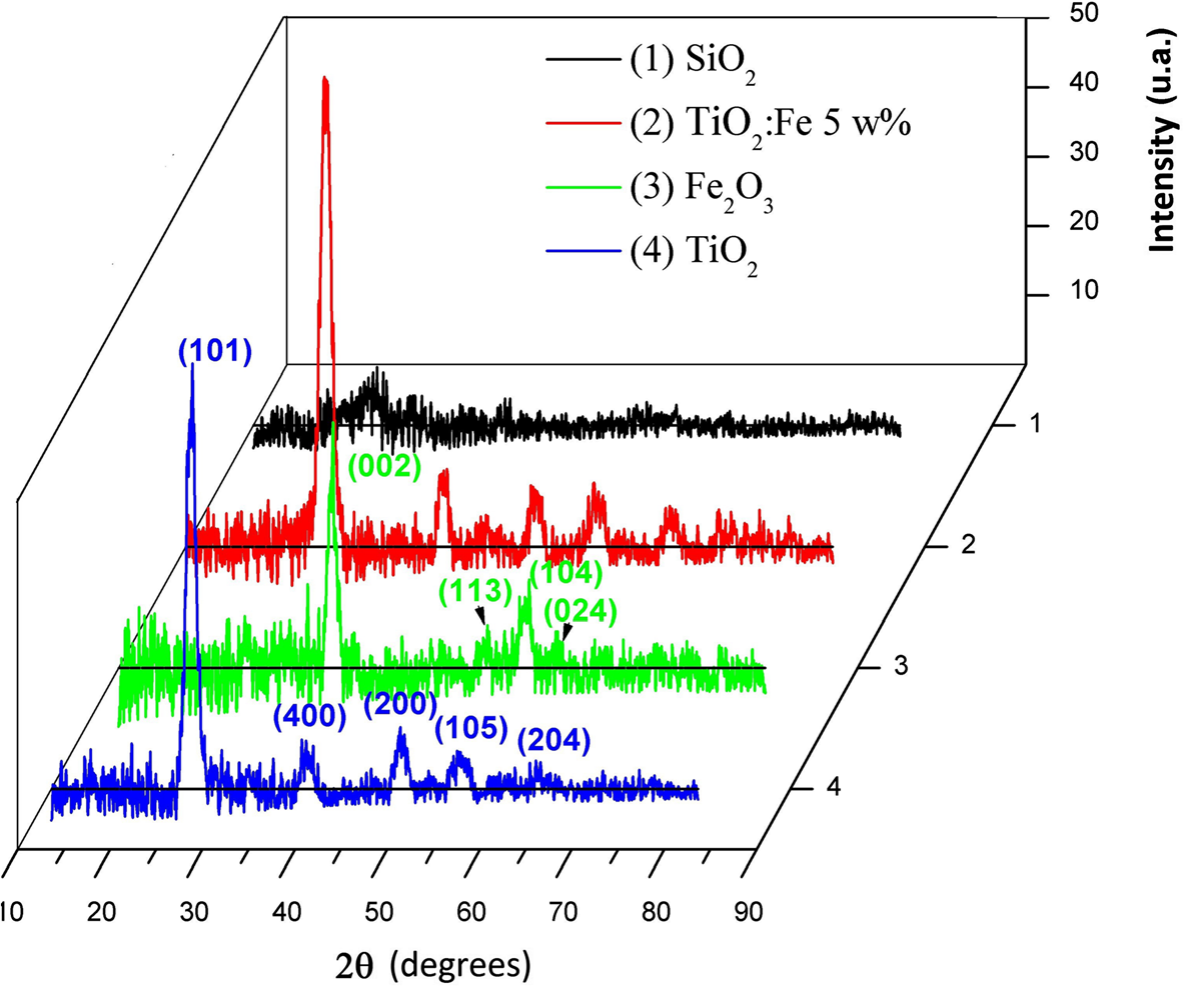
**X-ray diffractograms of the samples of SiO**_**2**_**, TiO**_**2**_**, Fe**_**2**_**O**_**3**_**, and TiO**_**2**_**:Fe**_**2**_**O**_**3 **_**5 wt%.**

**Figure 5:**
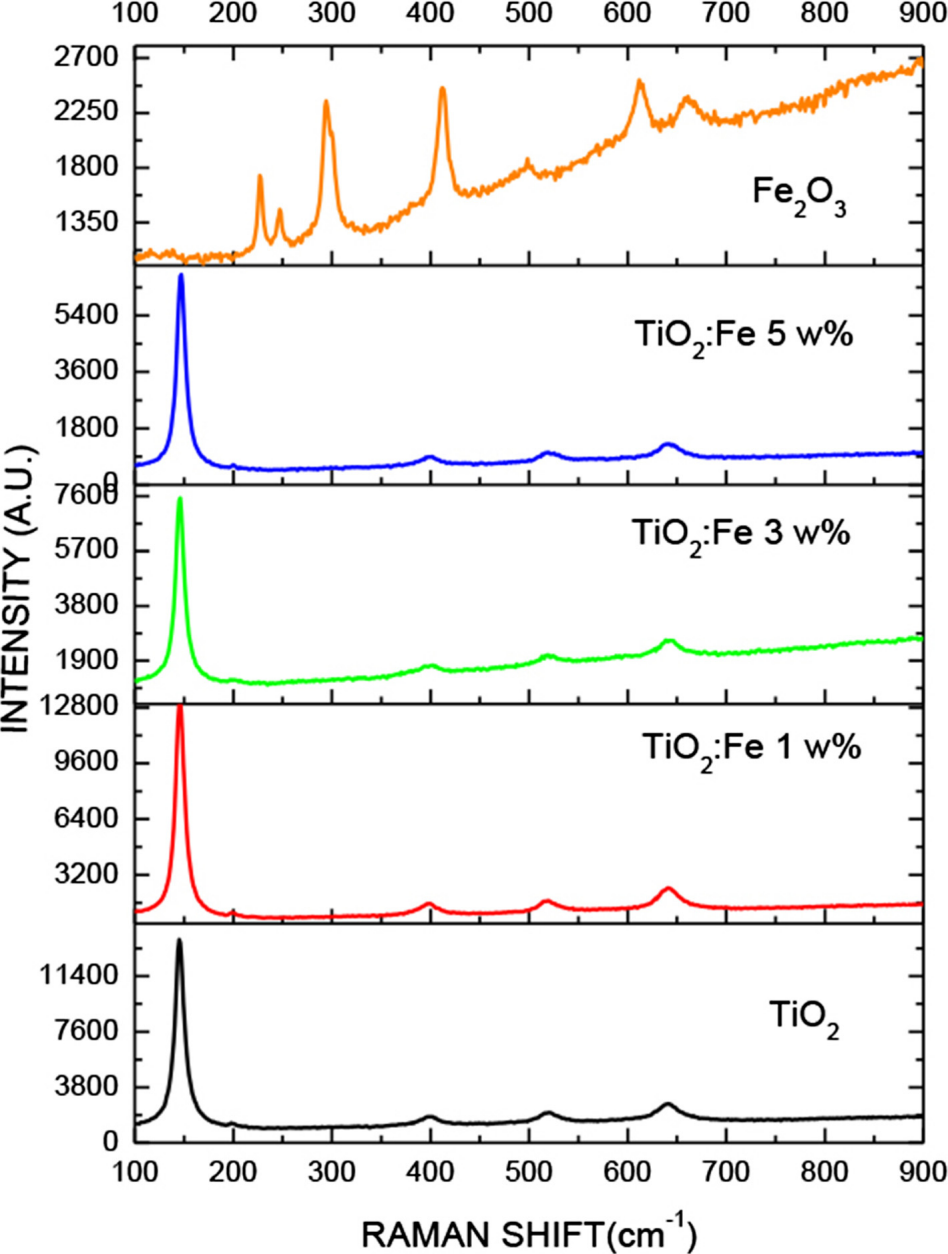
**μ-Raman spectra of the samples TiO**_**2**_**, Fe**_**2**_**O**_**3**_**, and TiO**_**2**_**:Fe**_**2**_**O**_**3 **_**composite at different Fe weight percentages.**

Figure 4 shows X-ray diffractograms of all prepared samples. The Fe_2_O_3_ X-ray diffractogram presents peaks corresponding to the alpha phase of this compound according to the JCPDS data card. It is observed that the peak intensity is enhanced as the Fe concentration is increased. The most intense peak appears at 28° and corresponds to the (101) plane of the anatase TiO_2_ structure. It is also observed that there is no significant effect of Fe on the TiO_2_ crystalline structure. However, there is a considerable effect of iron concentration on the TiO_2_ phonon modes of vibration as is shown in the μ-Raman spectra (see Figure 5).

The Raman lines located at 397, 522, and 642 cm^-1^ are, respectively, assigned to the B_1_, B_1g_, and A_1g_ vibration modes of TiO_2_. According to the analysis of group theory, the TiO_2_ anatase phase has six Raman active modes. The Raman spectrum of a TiO_2_ anatase single crystal has been investigated by Ohsaka et al. [8]; they have concluded that the six characteristic allowed modes appear at 146, 197, 400, 516, 520, and 641 cm^-1^.

The Raman lines appearing at 225, 245, 291, 410, 495, 611, and 1318 cm^-1^ are a characteristic of *α*-Fe_2_O_3_, i.e., the lines at 225 and 495 cm^-1^ are assigned to the A_1g_ vibration mode and the four peaks at about 245, 291, 410, and 611 cm^-1^ are attributed to the *E*_g_ vibration mode [9]. It should be noted that there is a line at 663 cm^-1^ which is typical for Fe_3_O_4_[10]. In our study, the Raman spectra of the TiO_2_ film supported on SiO_2_ opals are slightly shifted with respect to the TiO_2_ anatase phase obtained from TiO_2_ bulk; this shift could be due to the presence of Fe ions in the composite. In Figure 5, it is observed that the intensity of the phonon vibration mode at 149 cm^-1^ is decreased as the Fe^+3^ concentration is increased. These results suggest that when the Fe ions are incorporated into TiO_2_ crystal structure, species -Ti-O-Fe-O-Ti-O- type are formed. On the other side, in the Raman spectra of the TiO_2_:Fe_2_O_3_ composites, phonon lines corresponding to α-Fe_2_O_3_ are not observed.

**Figure 6:**
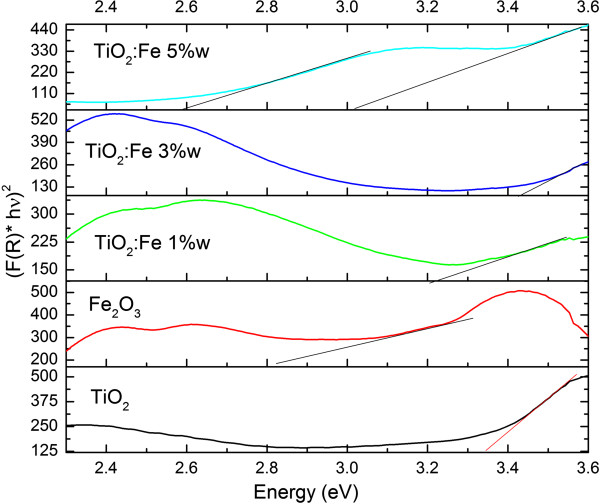
**Kubelka-Munk transformed reflectance spectra of the samples TiO**_**2**_**, Fe**_**2**_**O**_**3**_**, and TiO**_**2**_**:Fe**_**2**_**O**_**3 **_**at different Fe weight percentages.**

Reflectance spectra of the samples TiO_2_, Fe_2_O_3_, and TiO_2_:Fe_2_O_3_ composites at 1, 3, and 5 wt%, respectively, of Fe supported on the SiO_2_ microspheres are shown in Figure 6. The diffuse reflectance spectra were fitted using the Kubelka-Munk theory where the intersection of the fitted straight line and the photon energy axis gives the energy bandgap value [11,12]. Table 1 lists the energy bandgaps of TiO_2_, TiO_2_:Fe_2_O_3_ (1, 3, and 5 wt%), and Fe_2_O_3_ composites supported on SiO_2_ microsphere assemblies. It is seen that the energy bandgap values of TiO_2_:Fe_2_O_3_ composites lie between Eg = 3.5 eV of TiO_2_ bulk and Eg = 2.4 eV of Fe_2_O_3_. The energy bandgap values of the whole set of samples are listed in Table 1. This effect has been observed in a previous work [12].

**Table 1:** **Energy bandgap values of TiO**_**2**_**, Fe**_**2**_**O**_**3**_**, and TiO**_**2**_**:Fe**_**2**_**O**_**3 **_**at 1, 3, and 5 wt% composites supported on SiO**_**2 **_**microsphere assemblies**

**Samples**	**Eg (eV)**	***λ *** **(nm)**
TiO_2_	3.25	381
TiO_2_:Fe_2_O_3_ 1 wt%	2.79	444
TiO_2_:Fe_2_O_3_ 3 wt%	2.66	466
TiO_2_:Fe_2_O_3_ 5 wt%	2.53	489
Fe_2_O_3_	2.33	531

The decrement in energy bandgap of the TiO_2_-Fe_2_O_3_ composites with respect to the one of TiO_2_ bulk is probably due to the fact that Fe ions have been incorporated in the TiO_2_ lattice as indicated in the Raman section.

In the case of the Fe_2_O_3_ sample, we have observed at 2.44 eV a maximum which is considered to be the result of the pair excitation processes ^6^A_1_+ ^6^A_1_ → ^4^ T_1_(^4^G) + ^4^ T_1_(^4^G) [13]; in the case of 2.62 eV, other maximum in the reflection spectrum is observed due to the ^6^A_1_ → ^4^E, ^4^A_1_(^4^G) transition, in accordance to the Tanabe-Sugano diagram [13]. Here, we have also observed a reflection edge between 3.00 and 3.25 eV (413 and 381 nm), which could be due to the transition from the oxygen 2p orbital to the iron 3d orbital [14] or by the transition from the valence band to the conduction band of iron oxide. The bandgap value found for this oxide was 2.33 eV, which is a little greater than the one of reference value of Fe_2_O_3_ in bulk (2.22 eV). Additionally, there exists a maximum at 3.43 eV which is due to the contributions of the Fe^3+^ ligand field transitions ^6^A_1_ → ^4^E(^4^D) and ^6^A_1_ → ^4^ T_2_(^4^D) [13]. Otherwise, when the TiO_2_:Fe concentration is 1 and 3 wt%, the same maxima have been observed between 2.44 and 2.62 eV, which are related to the formation of Fe_2_O_3_ into the TiO_2_ lattice supported by the SiO_2_ microsphere assembly. However, the maximum at 3.43 eV disappears because more Fe ions (5 wt%) are introduced into the TiO_2_ lattice diminishing the energy bandgap of TiO_2_ which induces optical transitions in the visible region.

## Conclusions

An important effect of the Fe ion concentration on the morphological and optical properties of the TiO_2_ films supported on the single layer of SiO_2_ microsphere assemblies has been found. We have prepared silica microparticles of about 364 nm diameter by the Stöber method. We have been able to fabricate a single layer of SiO_2_ microspheres assembled in hexagonal close-packed fashion on glass substrate with a controlled vertical dip coating procedure.

We have observed that the TiO_2_ film formation affects the stability of the SiO_2_ layer cracking the layer and giving rise to the formation of ‘micro-shavings’ dispersed on the glass substrate, presenting a granular surface morphology with grain diameter of about 50 nm. The X-ray diffractograms do not show any significant effect of the Fe concentration into the TiO_2_ lattice structure. However, a considerable effect of iron concentration on the intensity of the TiO_2_ phonon vibration modes as shown in the μ-Raman spectra results has been observed. Furthermore, the diffuse reflectance spectra have shown that the energy bandgap of the TiO_2_:Fe_2_O_3_/SiO_2_ composites are located in the range between the energy bandgap of TiO_2_ and the one of Fe_2_O_3_ bulk.

## Competing interests

The authors declare that they have no competing interests.

## Authors’ contributions

AFPG and JIPF carried out the synthesis nanoparticle of SiO_2_. CMB carried out the studies of SEM and coordinated and helped to draft the manuscript. ESM participated in the project development and the experimental results of optical properties using spectrophotometry. EGB helped us in the interpretation of Raman results. FPR helped us in the interpretation of reflectivity spectra and participated in the project development. All authors read and approved the final manuscript.

## Authors’ information

C. Marquez-Beltran, Ph.D. is a professor research fellow at the Puebla University, Puebla, Mexico. The goal of his research is to master the nucleation and aggregation of nanoparticles in order to get a full morphological control in the production of nanostructured materials. J. I. Peña-Flores and A. F. Palomec-Garfias are graduate students. Estela Gómez-Barojas, Ph.D. is a researcher at the Puebla University, Puebla, Mexico. Her research area is synthesis and study of morphological and optical properties of semiconductors. Enrique Sanchez-Mora, Ph. D. is a researcher at the Puebla University, Puebla, Mexico. His research area is synthesis of metal oxides and their study of photocatalytic, chemical, and optical properties. Felipe Pérez-Rodríguez, Ph. D., is a researcher at the Puebla University, Puebla, Mexico. His research areas are physical properties of advanced materials and development of theoretical models to describe the phononic properties of metamaterials.
